# Differential expression of members of the E2F family of transcription factors in rodent testes

**DOI:** 10.1186/1477-7827-4-63

**Published:** 2006-12-05

**Authors:** Kame S El-Darwish, Martti Parvinen, Jorma Toppari

**Affiliations:** 1 Departments of Physiology and Pediatrics, University of Turku, Kiinamyllynkatu 10, FIN- 20520, Turku, Finland; 2 Department of Anatomy, University of Turku, Kiinamyllynkatu 10, FIN- 20520, Turku, Finland

## Abstract

**Background:**

The E2F family of transcription factors is required for the activation or repression of differentially expressed gene programs during the cell cycle in normal and abnormal development of tissues. We previously determined that members of the retinoblastoma protein family that interacts with the E2F family are differentially expressed and localized in almost all the different cell types and tissues of the testis and in response to known endocrine disruptors. In this study, the cell-specific and stage-specific expression of members of the E2F proteins has been elucidated.

**Methods:**

We used immunohistochemical (IHC) analysis of tissue sections and Western blot analysis of proteins, from whole testis and microdissected stages of seminiferous tubules to study the differential expression of the E2F proteins.

**Results:**

For most of the five E2F family members studied, the localizations appear conserved in the two most commonly studied rodent models, mice and rats, with some notable differences. Comparisons between wild type and E2F-1 knockout mice revealed that the level of E2F-1 protein is stage-specific and most abundant in leptotene to early pachytene spermatocytes of stages IX to XI of mouse while strong staining of E2F-1 in some cells close to the basal lamina of rat tubules suggest that it may also be expressed in undifferentiated spermatogonia. The age-dependent development of a Sertoli-cell-only phenotype in seminiferous tubules of E2F-1 knockout males corroborates this, and indicates that E2F-1 is required for spermatogonial stem cell renewal. Interestingly, E2F-3 appears in both terminally differentiated Sertoli cells, as well as spermatogonial cells in the differentiative pathway, while the remaining member of the activating E2Fs, E2F-2 is most concentrated in spermatocytes of mid to late prophase of meiosis. Comparisons between wildtype and E2F-4 knockout mice demonstrated that the level of E2F-4 protein displays a distinct profile of stage-specificity compared to E2F-1, which is probably related to its prevalence and role in Sertoli cells. IHC of rat testis indicates that localization of E2F-5 is distinct from that of E2F-4 and overlaps those of E2F-1 and E2F-2.

**Conclusion:**

The E2F-1 represents the subfamily of transcription factors required during stages of DNA replication and gene expression for development of germ cells and the E2F-4 represents the subfamily of transcription factors that help maintain gene expression for a terminally differentiated state within the testis.

## Background

**Figure 1:**
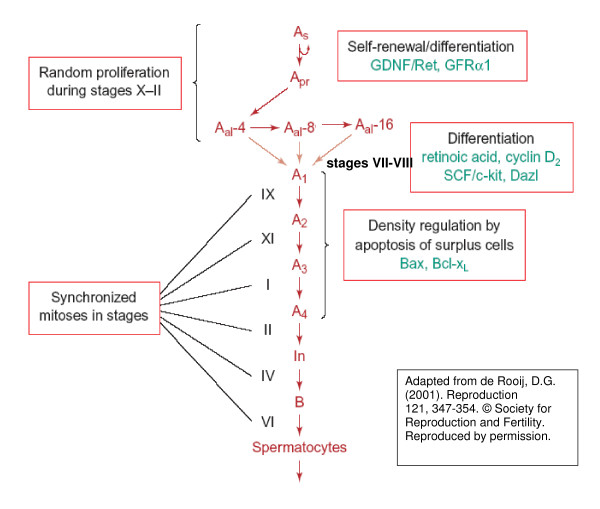
Stage-specificity of spermatogonial renewal, proliferation, differentiation, and its regulation, as reviewed by Dirk G. de Rooij [1]. Abbreviations: A_s _= spermatogonial stem cell, A_pr _= paired spermatogonia, A_al_-n = aligned spermatogonia of n number of cells, A_n _= differentiated type A spermatogonia entering synchronised division number n, In = intermediate type spermatogonia, B = B-type spermatogonia which undergo final mitotic division before replication and division for Meiosis I, GDNF/Ret are the ligand/receptor tyrosine kinase complex for glial cell-line derived neurotrophic factor (GDNF) signaling, GFRα1 is the glycosyl-phosphatidylinositol (GPI)-achored coreceptor for GDNF/Ret, SCF/c-kit are the ligand/receptor complex for stem cell factor (SCF) signaling, Dazl is the deleted in azoospermia-like gene, and Bax and Bcl-X_L _are pro-apoptopic and anti-apoptopic Bcl-2 protein family members respectively.

The testis provides an exceptional system for studying fate determination, differentiation, and death (apoptosis), interposed on mitosis and meiosis, and all these intertwined processes occurring in a single population of cells. The stages or fixed groups of cells undergoing different phases of spermatogenesis develop as a wave along the seminiferous tubule, continuously cycling over time. Spermatogonial stem cells are supposedly "awakened" from a quiescent state by signals such as glial cell line-derived neurotrophic factor (GDNF), and subsequent path selection maybe determined by the ratio of follicle stimulating hormone (FSH) to GDNF favoring differentiation over renewal, respectively [Fig. 1, adapted from [1]]. Subsequently, and concurrent with proliferation of type A spermatogonia committed to differentiation, stem cell factor (SCF) and retinoic acid are critical for survival and differentiation. Mitotic division and orientation away from the basement membrane, i.e. asymmetric mitosis, is also suggested to influence commitment to differentiation inasmuch as at least one daughter of an asymmetric mitotic division would be shifted away from the stem cell niche and towards the lower concentration of a gradient of signals, e.g. desert hedgehog, that repress differentiation [2]. Finally, in adult males, wherein the number of terminally differentiated Sertoli cells capable of supporting spermatogenesis has been established, normal sperm production, estimated at 1000/second in human [3], depends on the tight regulation of active proliferation, differentiation, and death [4].

The retinoblastoma protein (pRb) and related proteins p107 and p130 are key mediators of cell cycle arrest, differentiation, proliferation, senescence, and apoptosis, in response to a wide variety of signals. They fulfill their central role by interacting with a multitude of other proteins. The retinoblastoma protein is thought to interact with over 110 different partners [5], including transcription factors, to regulate the expression of genes affecting a cell's state of quiescence or differentiation, cycles of replication and division in active proliferation, or death. Most notable amongst pRb partners are the members of the E2F family of transcription factors. Different pRbs have preferential binding partners of the E2F family, and the expression of both partners is dependent on the stage of the cell cycle [6]. The pRbs are themselves tightly regulated by posttranslational modifications, especially phosphorylation at specific threonines and serines, catalyzed by cyclin dependant kinases (CDKs), which are regulated by cyclins. The levels of G1 cyclin D can be increased transcriptionally, translationally, or postranslationally by kinase-driven signal transduction pathways, which are activated upstream by signals that a cell receives, often referred to as the "context" of a cell. FSH and SCF are two of the key signals known to affect the differentiation of young (type A) spermatogonia, and both are known to affect an increase in cyclin D levels [7,8]. Since pRb is the principal substrate for cyclin D regulated CDKs 4 and 6, which initiate phosphorylation of pRb and its release of E2Fs 1–4, it is expected that the dynamic control of gene expression programs of spermatogenesis dependent on FSH and SCF be driven through interactions between the pRb and E2F families.

Our previous study of the retinoblastoma family of proteins (henceforth abbreviated pRbs) revealed differential expression and localization in the testis, indicating specialized roles for these cell cycle regulators and their partners in the development and maintenance of testis in rats [9,10]. Interestingly, the stage-specific expression pattern of pRb in Sertoli cells (highest at stages VII-VIII) disappears during days 2–20 after ethylene-dimethane sulfonate (EDS) treatment when testosterone levels are undetectable, suggesting that pRb levels can be affected by androgens. The level and phosphorylation status of p130 correlated with Leydig cell apoptosis: during the first days after EDS treatment, when massive Leydig cell apoptosis occurs, the level of p130 decreased and it became hypophosphorylated. Knockout mice have been invaluable to studies directed at determining the true biological roles of the pRbs, but reports of testis phenotypes have been conspicuously lacking. In contrast, knockout of E2F-1 produced testicular atrophy [11] with a Sertoli-cell-only (empty tubules) phenotype, and the addition of a hemizygous E2F-3 knockout to a full knockout of E2F-1 accelerates the development of testicular atrophy [12], even though knockout of E2F-3 alone exerts no effect on testis. This exacerbated phenotype in the compound or double knockout (DKO) suggested overlap in the biological roles of E2F-1 and E2F-3 in the testis. A knockout of an atypical member of the family, E2F-6, also generated a testis phenotype, manifesting Leydig cell hyperplasia and incomplete filling of epididymal ducts [13]. Knockout of E2F-4 causes infertility in both sexes, though the gonads appeared histologically normal [14,15]. These knockouts expressing testis phenotypes and the knockout of E2F-5 [16] and E2F-2 [17], which generated hydrocephaly and autoimmune phenotypes, respectively, underscore the critical role of E2Fs in regulating gene expression programs necessary for normal tissue differentiation and organogenesis. The study described herein was initiated in order to determine how the retinoblastoma and E2F families might interact to control the maintenance of testicular tissue organization, and the entry of undifferentiated quiescent spermatogonial cells into the mitotic proliferation leading to meiosis and differentiation into spermatozoa. The cell- and stage-specific localization of E2Fs in the testis from the study herein, compared to our previously developed map of the pRbs in testis [9,10], correlates with traditional paradigms of partnerships between different family members and their roles in regulating the expression of programs of genes required for active proliferation versus differentiation. However, some of our results may also suggest a more independent role for certain members or unexpected partnerships between these two protein families.

## Materials and methods

The E2F-1 -/- mice [18], B6;129S4-E2f1tm1Meg/J, and control mice (B6129SF2/J) were purchased from the Jackson Laboratory. Patrick Humbert generously provided the E2F-4 -/- mice [14] and E2F-5/mice [16]. FVB/N mice were obtained from breeding stocks of the Transgenic Core Facility and Sprague-Dawley rats were obtained from breeding stocks of the Central Animal Laboratory of the University of Turku. All procedures performed on animals were in strict accordance with the European Convention for the Protection of Vertebrate Animals used for Experimental and other Scientific Purposes (ETS No.123), Appendix A: Guidelines for accommodation and care of animals, and the European Union Directive 86/609/ETY on the protection of animals used for experimental and other scientific purposes.

For the immunohistochemical analysis of E2Fs, sections of paraformaldehyde-fixed and paraffin-embedded (FFPE) testis were deparaffinized and rehydrated as described elsewhere [19]. For antigen retrieval, slides were incubated in 10 mM citrate buffer pH 6.0 heated to boiling in a microwave oven, for ten minutes [20,21]. Staining with primary monoclonal antibodies, counterstaining with horseradish peroxidase (HRP)-conjugated anti-mouse IgG secondary antibody, and the staining with peroxidase substrate were performed using the PowerVision+ and Homomouse IHC kits and protocols from ImmunoVision Technologies, Co. The primary antibodies used for immunohistochemistry were mouse monoclonal antibodies (MAbs) of the same immunoglobulin isotype and light chain against E2F-1, E2F-2 and E2F-4, and a synthetic hapten which is normally not present animals (negative control) purchased from Lab Vision Corporation, a mouse monoclonal antibody against E2F-3 from Upstate, a mouse monoclonal antibody against E2F-5 from Santa Cruz Biotechnology, Inc., rabbit polyclonal antibodies against p107, SMADs (small mothers against decapentaplegic) 1/2/3, SMAD 4 and androgen receptor (positive control) from Santa Cruz Biotechnology, Inc., and a rabbit polyclonal antibody against a synthetic hapten which is normally not present in animals (negative control) from Lab Vision Corporation. Staining with negative control antibodies was always performed with at least the same dilution as the least dilute test antibody. Immunohistochemistry results were collated from repeated analyses of sections of testis from at least three individuals of each species, rat and mouse testes sections stained at the same time, and sections of testes from at least three pairs of littermates or age-matched (8 weeks or older) individuals of wild type and knockout mice from each line stained at the same time. The primary antibodies used for Western blot analyses were MAbs against E2F-1 and E2F-4 from Lab Vision Corporation, a MAb against E2F-4 provided by Jacqueline Lees, and a MAb against glyceraldehyde-3-phosphate dehydrogenase (GAPDH) from HyTest Ltd. The HRP-conjugated secondary antibodies used in Western blot analysis were purchased from Amersham Biosciences.

Segments containing defined stages of mouse seminiferous tubule were microdissected from the testis of adult animals, on a transilluminating stereomicroscope [22] and flash-frozen in liquid nitrogen. Total proteins were extracted by homogenizing frozen tubules directly into an extraction buffer (Cell Cycle Methods booklet 4, Biosource International) containing a cocktail of protease inhibitors (complete Mini, from Roche Diagnostics GmbH) and activated orthovanadate (protocol from Upstate), briefly vortexing to facilitate thawing, and passaging 6–8 times through a 25G 5/8" needle using a 1 ml syringe. The resulting extracts were incubated at room temperature for 15 min, before centrifugation at 16 000 g for 4°C. Then the extracts were kept on ice if the concentrations of protein were to be determined right away or flash-freeze in liquid nitrogen before storing at -80°C. The concentrations of protein in different extracts were quantitated by bicinchoninic acid (BCA) assay from Pierce. One hundred micrograms samples of the protein extracts were resolved through sodium dodecyl sulfate polyacrylamide gel electrophoresis (SDS-PAGE), with a 4% acrylamide stacking gel and 8% acrylamide resolving gel. Western transfer to a polyvinylidene fluoride (PVDF) membrane was performed as described elsewhere [23], except that ethanol was used in place of methanol in the transfer buffer. After immunostaining with primary and secondary antibodies, bands were detected on blots by treatment for enhanced chemiluminescent (ECL) detection with the enhanced ECL plus kit (Amersham-Biosciences) and exposure on Fuji Rx 100 X-ray film. The level of GAPDH protein, i.e. stained band intensity, was used as a control on immunostained Western blots, for quantitation of total proteins by BCA assay, and for loading and transfer of total proteins in SDS-PAGE and electrophoretic transfer.

The developed films were scanned on a UMAX Astra 2000U and densitometric analysis of specific bands was performed with the Image J (1.37u) software by Wayne Rasband, the National Institutes of Health, USA. Data from at least three independent experiments were processed for statistical analysis of variance (ANOVA) for repeated measures by the WINKS 4.80a software from TexaSoft, USA. Using the Newman-Keuls multiple comparisons test, the means of relative levels of E2F for different pooled stage(s) were not considered significantly different at the 0.05 significance level, while differences between the means were judged as significant at a p < 0.025, and plots of the means reflect these groupings.

## Results

### Localizations of E2F-1, E2F-2 and E2F-3

**Figure 2:**
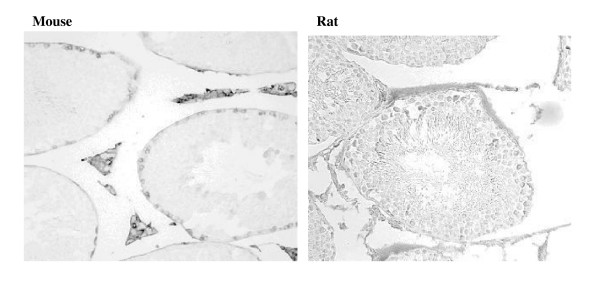
Immunohistochemical staining of sections from testis of mouse and rat with antibody against synthetic negative control peptide.

**Figure 3:**
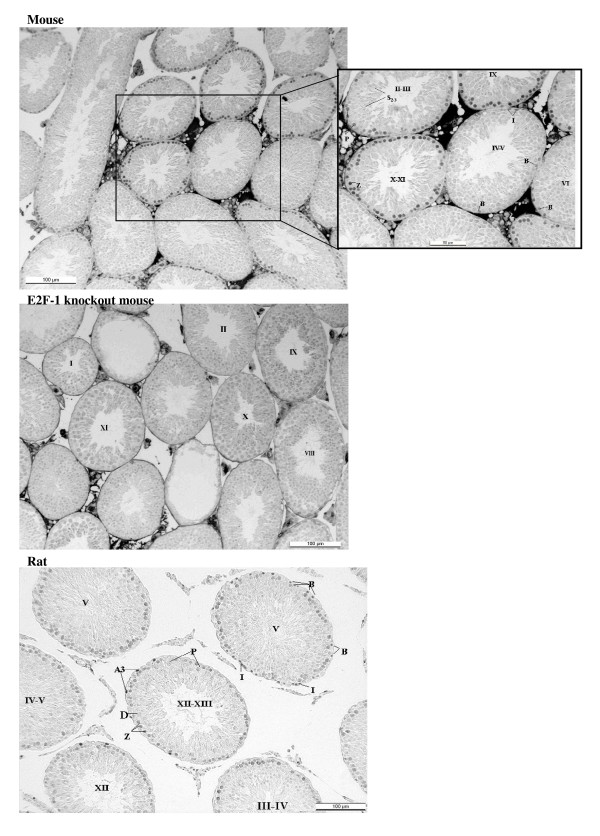
Immunohistochemical staining of sections from testis of mouse, E2F-1 knockout mouse, and rat with antibody against E2F-1. Labels are: for germline type A spermatogonia (A), intermediate (I) and B-type (B) spermatogonia, preleptotene (Pl), pachythene (P), zygotene (Z), and diplotene (D)spermatocytes, spermatids (s), and for somatic Sertoli cell (S), Leydig cell (L), and peritubular myoid cell (M); stages indicated by Roman numerals I-XIV for rat and I-XII for mouse.

**Figure 4:**
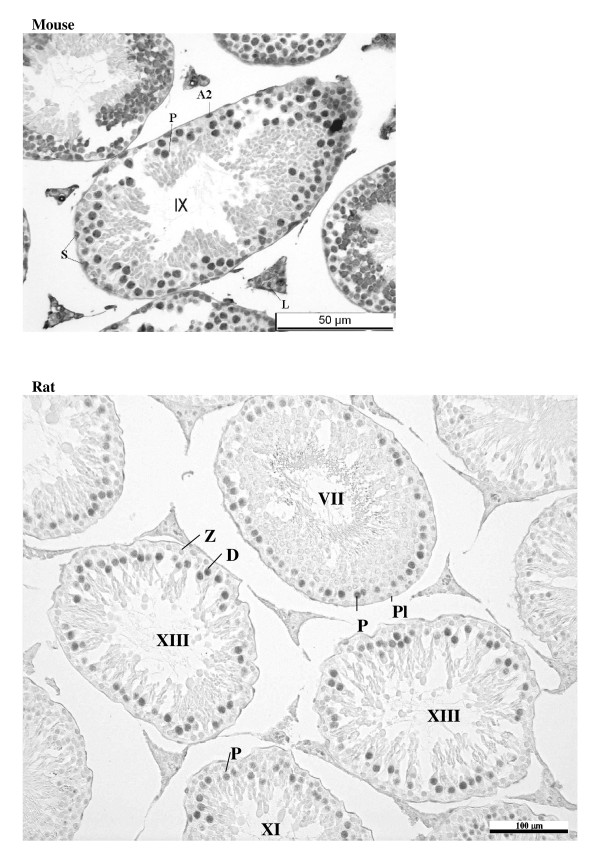
Immunohistochemical staining of sections from testis of mouse and rat with antibody against E2F-2. Labels are: for germline type A spermatogonia (A), intermediate (I) and B-type (B) spermatogonia, preleptotene (Pl), pachythene (P), zygotene (Z), and diplotene (D)spermatocytes, spermatids (s), and for somatic Sertoli cell (S), Leydig cell (L), and peritubular myoid cell (M); stages indicated by Roman numerals I-XIV for rat and I-XII for mouse.

**Figure 5:**
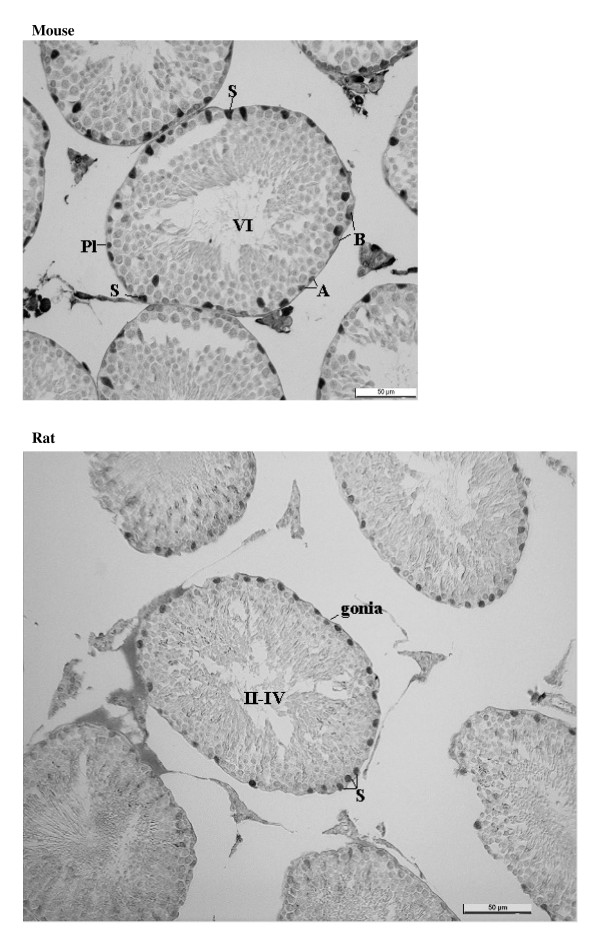
Immunohistochemical staining of sections from testis of mouse and rat with antibody against E2F-3. Labels are: for germline type A spermatogonia (A), intermediate (I) and B-type (B) spermatogonia, preleptotene (Pl), pachythene (P), zygotene (Z), and diplotene (D)spermatocytes, spermatids (s), and for somatic Sertoli cell (S), Leydig cell (L), and peritubular myoid cell (M); stages indicated by Roman numerals I-XIV for rat and I-XII for mouse.

Staining for E2F-1 appears to be somewhat different for rat compared to mouse testis (Fig. 3), the IHCs have in common the staining of nuclei of spermatocytes from leptotene to early pachytene, most evident in stages XI to XI. The specificity of staining by the anti-E2F-1 MAb on wildtype mouse sections was established by simultaneously staining these sections alongside sections from an E2F-1 -/- (knockout) (Fig. 3), wherein hardly any staining is visible above that obtained for the negative control MAb (Fig. 2). Staining of rat sections with anti-E2F-1 also reveals some type A3 spermatogonia in stages XIII, as well as type A and intermediate type spermatogonia in stages II to V, and IX (Fig. 3, and Fig. 11). These observations from immunohistochemistry for E2F-1 are in concordance with the results from Western blot analyses that displayed a most distinct increase in the level of a 56 kDa protein corresponding to E2F-1, which was absent from separated sample of E2F-1 knockout testis (data not shown), between stages VIII and IX of mouse (Fig. 16). E2F-2 is localized most abundantly in the nuclei of pachytene and diplotene spermatocytes of stages VII-XIII, but also lighter staining of secondary spematocytes at stage XIV of rat is apparent (Fig. 4 and Fig. 12). E2F-3 is most strongly stained in the nuclei of type A, intermediate and type B spermatogonia of stages II to V of rat, with lighter staining in Sertoli cells of other stages (Fig. 5, and Fig. 13). The antibody against E2F-3 stained Sertoli cells in mouse testis sections more strongly than on rat testis sections.

### Differential compartmentalization of E2F-4 and E2F-5

**Figure 6:**
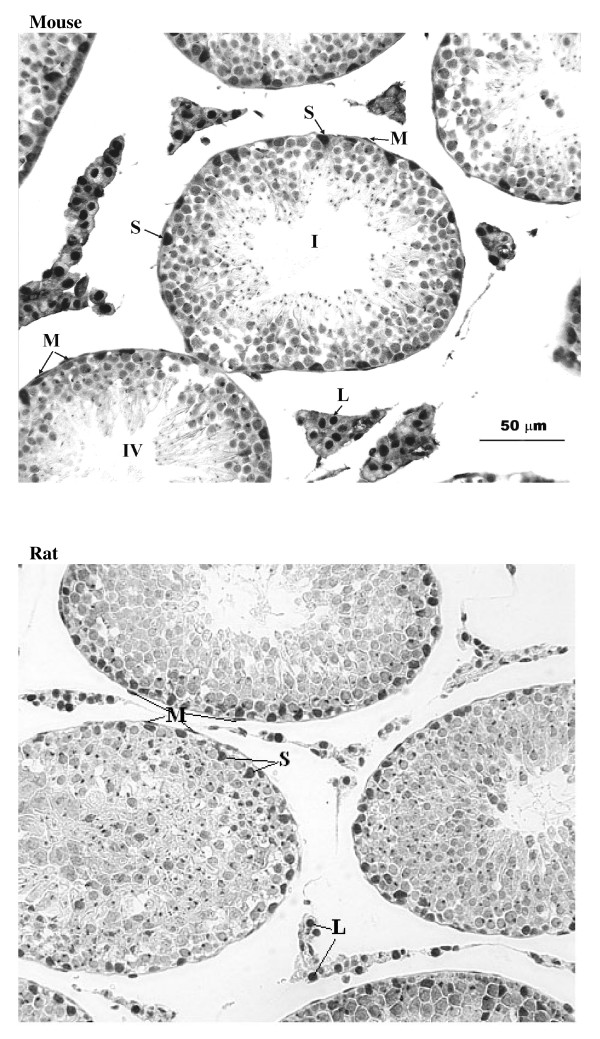
Immunohistochemical staining of sections from testis of mouse and rat with antibody against E2F-4. Labels are: for germline type A spermatogonia (A), intermediate (I) and B-type (B) spermatogonia, preleptotene (Pl), pachythene (P), zygotene (Z), and diplotene (D)spermatocytes, spermatids (s), and for somatic Sertoli cell (S), Leydig cell (L), and peritubular myoid cell (M); stages indicated by Roman numerals I-XIV for rat and I-XII for mouse.

**Figure 7:**
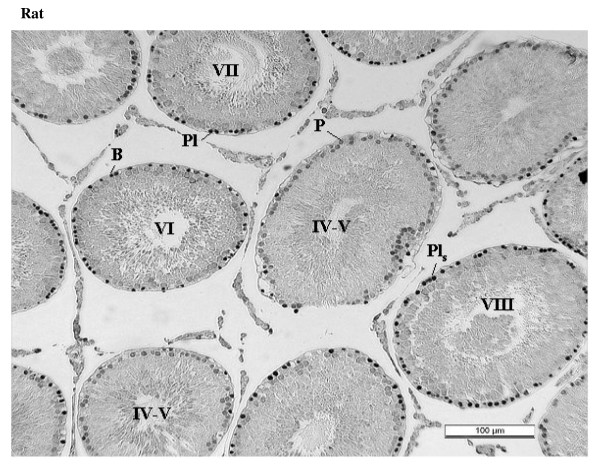
Immunohistochemical staining of section from testis of rat with antibody against E2F-5. Labels are: for germline type A spermatogonia (A), intermediate (I) and B-type (B) spermatogonia, preleptotene (Pl), pachythene (P), zygotene (Z), and diplotene (D)spermatocytes, spermatids (s), and for somatic Sertoli cell (S), Leydig cell (L), and peritubular myoid cell (M); stages indicated by Roman numerals I-XIV for rat.

**Figure 8:**
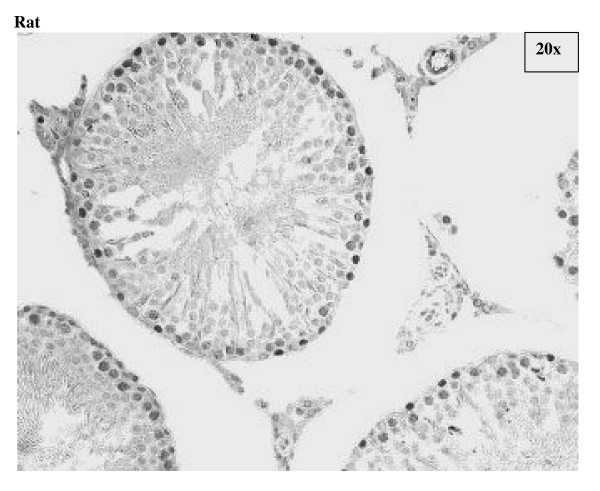
Immunohistochemical staining of section from testis of rat with antibody against p107.

**Figure 9:**
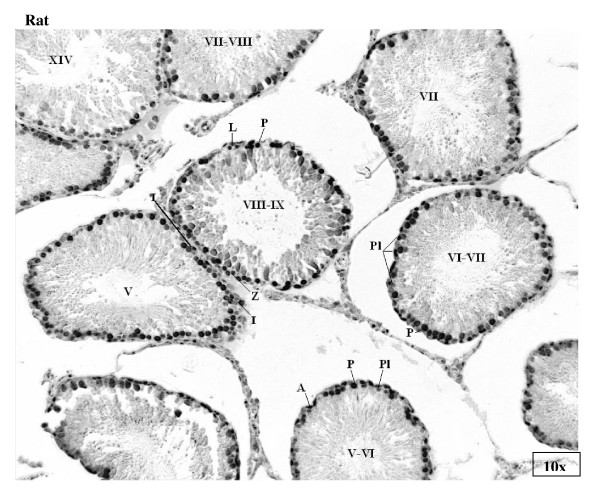
Immunohistochemical staining of section from testis of rat with antibody against SMADs 1/2/3. Labels are: for germline type A spermatogonia (A), intermediate (I) and B-type (B) spermatogonia, preleptotene (Pl), pachythene (P), zygotene (Z), and diplotene (D)spermatocytes, spermatids (s), and for somatic Sertoli cell (S), Leydig cell (L), and peritubular myoid cell (M); stages indicated by Roman numerals I-XIV for rat.

**Figure 10:**
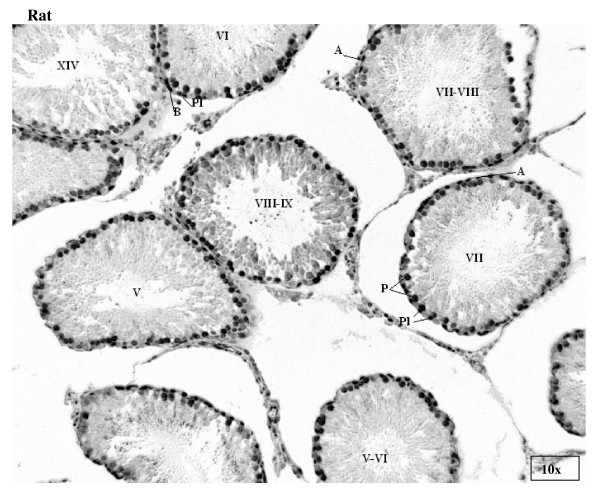
Immunohistochemical staining of section from testis of rat with antibody against SMAD 4. Labels are: for germline type A spermatogonia (A), intermediate (I) and B-type (B) spermatogonia, preleptotene (Pl), pachythene (P), zygotene (Z), and diplotene (D)spermatocytes, spermatids (s), and for somatic Sertoli cell (S), Leydig cell (L), and peritubular myoid cell (M); stages indicated by Roman numerals I-XIV for rat.

The antibody against E2F-4 stained the nuclei of the somatic cells (Leydig, Sertoli and peritubular myoid cells) of the testis most strongly, and especially so the Leydig cells. However, parallel staining of testis sections from E2F-4 knockout mice demonstrated that only staining of Sertoli cells was absent (data not shown). The staining of Sertoli cells appears darker in stages IV to VI than in other stages (Fig. 6, and Fig. 14). The antibody used to detect E2F-5 in the rat testis, where it most intensely stained nuclei of type B spermatogonia, preleptotene and pachytene spermatocytes in stages from IV to VIII (Fig. 7, and Fig. 15) did not stain sections of wild type and E2F-5 knockout mouse testis specifically under any of the conditions tested, in parallel experiments. At higher magnification, staining of rat testis sections with anti-E2F-5 appears to display a perinuclear or diffuse cytoplasmic staining in zygotene spermatocytes of stage XIII to late pachytene of stage VII. Staining for p107 appears most prevalent in differentiating spermatogonia, and lesser in early spermatocytes in meiosis (Fig. 8), while stainings for SMADs 1/2/3 (R-SMADs) and SMAD 4 (N-SMAD) revealed strongest localization of these in spermatogonia and spermatocytes up to the pachytene stage, but not beyond (Figures 9 and 10).

**Figure 11:**
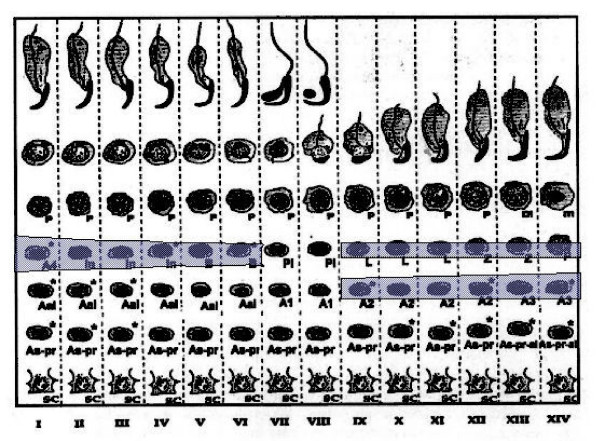
Chart of immunohistochemical staining of E2F-1, in cells and stages of rat seminiferous tubule cycle. The width of shading indicates intensity of staining. Abbreviations: As = spermatogonial stem cell, As-pr = paired spermatogonia, As-pr-al = paired and aligned spermatogonia, Aal-n = aligned spermatogonia of n number of cells, An = differentiated type A spermatogonia entering synchronised division number n, In = intermediate type spermatogonia, B = B-type spermatogonia which undergo final mitotic division before replication and division for Meiosis I, Pl = preleptotene, L = leptotene, Z = zygotene, P = pachythene, m = meiosis, In = intermediate, and Sc = Sertoli cell.

**Figure 12:**
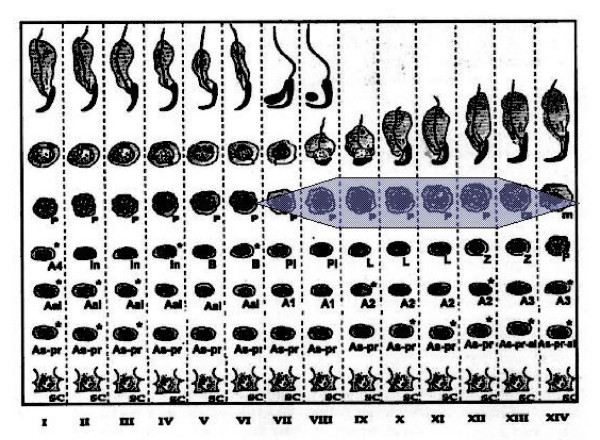
Chart of immunohistochemical staining of E2F-2, in cells and stages of rat seminiferous tubule cycle. The width of shading indicates intensity of staining. Abbreviations: As = spermatogonial stem cell, As-pr = paired spermatogonia, As-pr-al = paired and aligned spermatogonia, Aal-n = aligned spermatogonia of n number of cells, An = differentiated type A spermatogonia entering synchronised division number n, In = intermediate type spermatogonia, B = B-type spermatogonia which undergo final mitotic division before replication and division for Meiosis I, Pl = preleptotene, L = leptotene, Z = zygotene, P = pachythene, m = meiosis, In = intermediate, and Sc = Sertoli cell.

**Figure 13:**
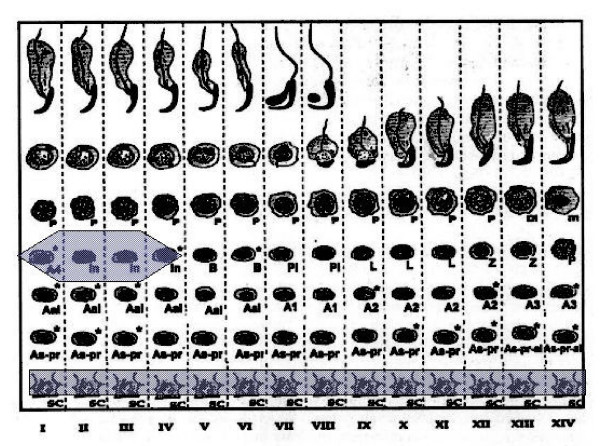
Chart of immunohistochemical staining of E2F-3, in cells and stages of rat seminiferous tubule cycle. The width of shading indicates intensity of staining. Abbreviations: As = spermatogonial stem cell, As-pr = paired spermatogonia, As-pr-al = paired and aligned spermatogonia, Aal-n = aligned spermatogonia of n number of cells, An = differentiated type A spermatogonia entering synchronised division number n, In = intermediate type spermatogonia, B = B-type spermatogonia which undergo final mitotic division before replication and division for Meiosis I, Pl = preleptotene, L = leptotene, Z = zygotene, P = pachythene, m = meiosis, In = intermediate, and Sc = Sertoli cell.

**Figure 14:**
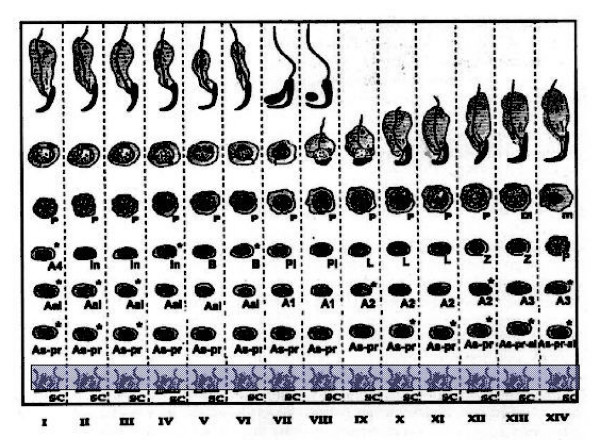
Chart of immunohistochemical staining of E2F-4, in cells and stages of rat seminiferous tubule cycle. The width of shading indicates intensity of staining. Abbreviations: As = spermatogonial stem cell, As-pr = paired spermatogonia, As-pr-al = paired and aligned spermatogonia, Aal-n = aligned spermatogonia of n number of cells, An = differentiated type A spermatogonia entering synchronised division number n, In = intermediate type spermatogonia, B = B-type spermatogonia which undergo final mitotic division before replication and division for Meiosis I, Pl = preleptotene, L = leptotene, Z = zygotene, P = pachythene, m = meiosis, In = intermediate, and Sc = Sertoli cell.

**Figure 15:**
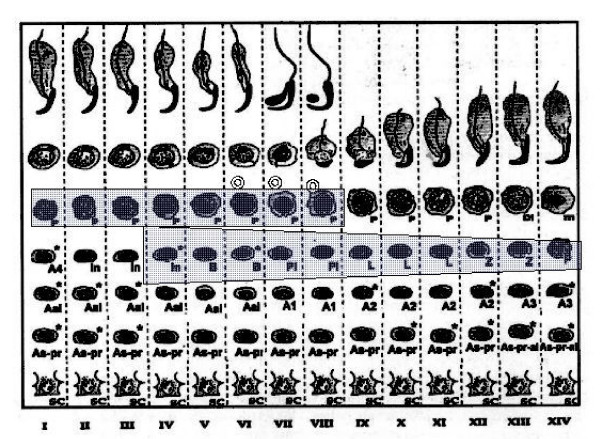
Chart of immunohistochemical staining of E2F-5, in cells and stages of rat seminiferous tubule cycle. The width of shading indicates intensity of staining. Abbreviations: As = spermatogonial stem cell, As-pr = paired spermatogonia, As-pr-al = paired and aligned spermatogonia, Aal-n = aligned spermatogonia of n number of cells, An = differentiated type A spermatogonia entering synchronised division number n, In = intermediate type spermatogonia, B = B-type spermatogonia which undergo final mitotic division before replication and division for Meiosis I, Pl = preleptotene, L = leptotene, Z = zygotene, P = pachythene, m = meiosis, In = intermediate, and Sc = Sertoli cell.

Western blot analyses demonstrated a 65 kDa protein corresponding to E2F-4 (Paul Danielen, MIT, personal communication) present in samples extracted from wild type (E2F-4 +/+) and heterozygous (E2F-4 +/-) mouse testis, but absent in protein samples from testis of E2F-4 knockout siblings, separated in parallel. The level of the E2F-4 band increased between stages VI and VII, and then plateaued over remaining stages of the seminiferous epithelium (Fig. 17). It was not possible to detect specifically E2F-5 protein in wild type compared to E2F-5 knockout mouse testis. Nevertheless, Western blot analyses of protein extracts from ten pooled stages of microdissected rat testis stained with antibody for detescting E2F-5 detected a protein about 65 kDa band that peaked between stages II-III and IV-V just before the stageVI transition of the seminiferous epithelium (data not shown). Preliminary RT-PCR results (not shown) for E2F-5 demonstrate its transcript level is constant throughout the cycle of the seminiferous epithelium.

**Figure 16:**
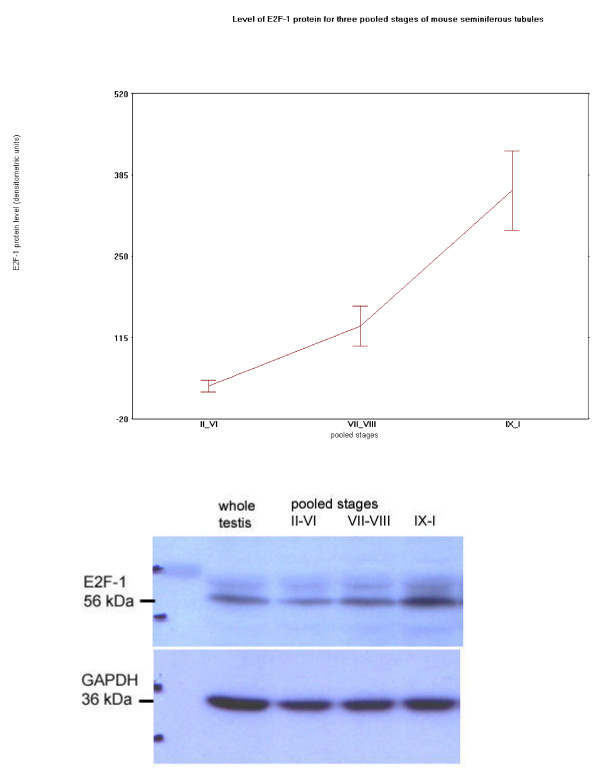
Plot of specific protein level, and immunostained Western blot representative of four different sets, for E2F-1, and GAPDH, in total proteins extracted from three pooled stages of mouse.

**Figure 17:**
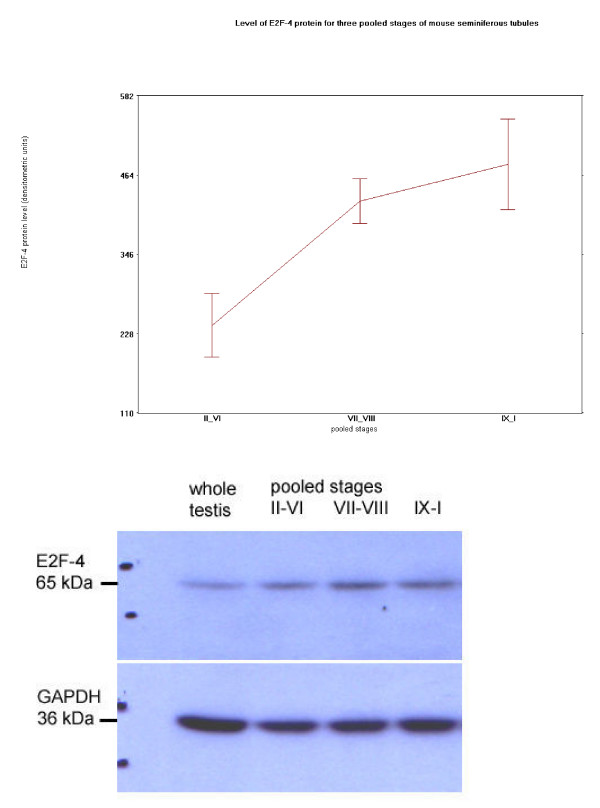
Plot of specific protein level, and immunostained Western blot representative of four different sets, for E2F-4, and GAPDH, in total proteins extracted from three pooled stages of mouse.

## Discussion

### E2F-1

The detection of E2F-1 in early (type A) spermatogonial cells at stages II-V of rat testis but not mouse testis sections may be attributable to greater homology of the epitope (residues 342–386 of the human sequence) used to raise the KH-129 monoclonal antibody to the corresponding rat sequence. Anyway, very early type A spermatogonia, that might represent true spermatogonial stem cells (SSCs), are notoriously elusive to IHC staining. Still, the observation of Sertoli-cell-only tubules in E2F-1 -/- indicates that E2F-1 is likely expressed and activating gene programs required for proliferation in renewal as well as differentiation of SSCs. Other immunohistochemical studies conducted in our lab on sections from excised human testicular tumors have demonstrated that E2F-1 is strongly expressed in carcinoma *in situ *(data not submitted), supporting its role in initiating proliferation of SSCs in testis.

Staining of leptotene to early pachytene spermatocytes at stages IX to XI of wildtype mouse, and the conservation of such a staining pattern in the rat indicate that these cells are most likely to require E2F-1 regulated gene expression for the transition through prophase of meiosis I. It is conceivable that E2F-1 might also be responsible for activating the expression of pro-apoptosis genes in these cell types when stabilized by checkpoint proteins in the response to DNA double-strand breaks caused by genotoxic insult. In fact, apoptosis is mostly observed in the very cell types and stages in which we observe most E2F-1 in the testis. Such duality in the role of E2F-1 in the testis might explain why its level appears diminished in "runaway" seminomatous tumours of testis (data not submitted).

### E2F-2

The observation of E2F-2 expression in cells wherein p107 had also been localized to a lesser extent (fig. 8), and wherein pRb was absent, is somewhat unexpected. However, others have observed that G1 CDKs can phosphorylate p107 to an extent which increases its binding to E2F-1 at least transiently [24], though p107 otherwise displays a preference for binding E2F-4. Perhaps site-specific phosphorylation of p107 by cyclin A1 [25] or cyclin E2 [26] and cdk2 [27] enables it to interact with E2F-2 during meiosis. Alternatively, E2F-2 might be acting independently of p107 at these specific stages of spermatogenesis. Nevertheless, E2F-2 -/- mice were fertile [17] and no abnormal histology of testis was reported for the E2F-2 knockout or E2F-2/E2F-1 DKO mice. This indicates that another member of the E2F family might be able to compensate at this stage.

### E2F-3

The prevalence of E2F-3 in spermatogonia of stages II to IV, and Sertoli cells of other stages, is especially noteworthy in light of the observation by others that E2F-1 -/- E2F-3 +/- DKO mice display an accelerated atrophy in testis development compared to just E2F-1 -/- [12] However, staining of spermatogonia might also be explained by possible cross-reactivity of the monoclonal antibody used to detect E2F-3 for E2F-1. Compound E2F-1 -/- E2F-3 +/- DKO males have severely atrophied testis and tubules already at 4 months (120 days) of age. The phenotype observed for the DKO indicates E2F-1 is indispensable for spermatogonial stem cell renewal. Our observation of E2F-3 in Sertoli cells might explain how a malfunction of Sertoli cells in the DKO could exacerbate the E2F-1 phenotype, since proper Sertoli cell function is necessary for normal maintenance of the testis. Combinatorial interactions between the functionalities of E2F-1 and E2F-3 have been recently characterized [28].

### E2F-4

The localization of E2F-4 to the same somatic cell compartment, the Sertoli cells, as p130 was previously mapped to agrees with the paradigm of its role in maintaining terminally differentiated states by repressing the expression of genes expressed in cycling cells. Furthermore, the increasing level of E2F-4 protein detected from stages VII on could relate to the testosterone sensitivity of Sertoli cells during this period of the seminiferous epithelial cycle.

### E2F-5

**Figure 18:**
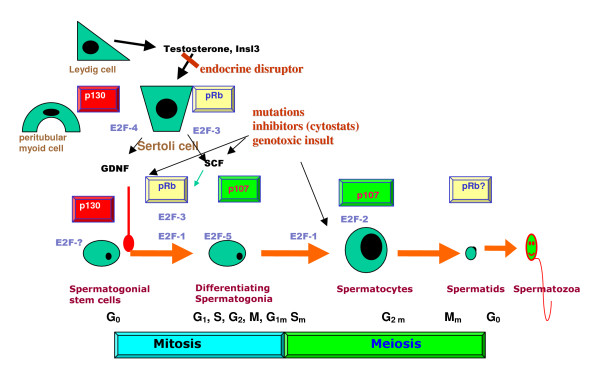
Diagram summarizing differential expression of members of the retinoblastoma proteins [9, 10] and their E2F partners in testis, and showing how endocrine signals and other factors could influence testis development via interactions between these two families of cell cycle regulators.

This localization of E2F-5 resembles that of p107 (Fig. 8), which was determined from earlier studies (Fig. 18) to be expressed throughout meiosis. Furthermore, when compared to the staining of young primary spermatocytes with antibodies against the SMADs (Figures 9 and 10), it is possible to envision how E2F-5 might interact with p107, activated SMADS 2/3 and SMAD 4 to be tranlocated to the nucleus [29] in leptotene, zygotene and early pachytene spermatocytes of stages VI to XIV. Such a tripartite complex interaction has been recently demonstrated for TGF-beta mediated repression of replication of cells in culture [30]. However, the IHC of rat testis displaying a localization of E2F-5 distinct from that of E2F-4 and overlapping those of E2F-1 and E2F-2 might also be explained by a possibility of cross-reactivity of the monoclonal antibody used to detect E2F-5 with the E2F-1 and E2F-2 of rat, since we were not able to detect any significant signals from staining of mouse testis sections.

### Distinct localizations of E2F-1 and E2F-4

This observation fits nicely with the scheme of spermatogonial development presented in Figure 1, since E2F-1 is considered a key transcription factor capable of transactivating the expression of numerous proteins required for DNA replication and the transition to S phase of the cell cycle in actively proliferating cells. E2F-1 is also somewhat unique in its dual capacity to transactivate the expression of pro-apoptotic genes in response to DNA-damage-activated checkpoints at the S/G2 transition, and perhaps this dualism in E2F-1's roles is related to the observation that apoptosis of spermatogonia coincides (Fig. 1) with the stages of peak E2F-1 levels. In contrast, E2F-5 might be expected to repress the expression of S phase genes [31], while phosphorylated E2F-5 could function as a coactivator and autoregulator of late G1 and S phase genes as observed by others [32], perhaps transactivating the expression of the meiosis-specific Cyclin E2 or Cyclin A, and possibly other genes required for meiosis and differentiation [16,33].

## Conclusion

The observations described above indicate a scenario (Fig. 18) wherein E2F-1 is expressed in very young type A spermatogonial (stem) cells, suggesting that E2F-1 is a key transcription factor in activating the expression of genes necessary for entering the proliferative phase of spermatogenesis. This might follow p130 phosphorylation and inactivation in response to paracrine GDNF secretion from Sertoli cells stimulated by the endocrine gonadotropin FSH. It is quite possible that pRb levels increase at this point in order to maintain a check on E2F-1 and its pro-apoptotic activities. That E2F-1, E2F-5 and E2F-2 are the principle members of the E2F family present in germ cells during meiosis, alongside our previous observation that the only member of the retinoblastoma protein family to be expressed throughout this period is p107 suggests that these three E2Fs are interacting with p107 in order to target its repressive role to specific genes. Otherwise, they could be acting independently of a retinoblastoma protein in regulating the expression of different sets of genes at different stages of the extended prophase of meiosis. E2F-3 appears to be serving dual roles in Sertoli cells and late spermatogonia, most likely under the hormone-responsive control of pRb. This later observation presents the tantalizing prospect that E2F-3 might actually be interacting with the androgen receptor (AR) of Sertoli cells via its Rb partner, since it has been previously demonstrated by others that pRb can serve a coactivator role in complexes with AR. In germ cells, E2F-3 appears to be filling in a gap between E2F-1 and E2F-5 at the intermediate stage between type A and type B spermatogonia, possibly activating the expression of genes required for commitment to the differentiative pathway, following inactivation of pRb in response to the SCF ligand. In Sertoli cells, E2F-3 is most likely checked by pRb, but then released and activating the expression of genes in a signal transduction cascade response to stimulation by gonadotropins and testosterone. It is conceivable that there is an overlap of target genes controlled by E2F-3 and E2F-4 in Sertoli cells. This could be resolved at least in part by ongoing chromatin immunoprecipitation for genomic microarray (ChIP-on-chip) studies to ascertain the target genes of E2Fs expressed in testis. Another key question is the identity of the E2F that interacts with p130 in quiescent spermatogonial stem cells.

## Competing interests

The author(s) declare that they have no competing interests.

## Authors' contributions

KED designed the study, performed all the experiments, and wrote and revised all drafts of the manuscript. MP helped with microdissection, and provided comments to results and drafts of the manuscript. JT provided background information on testis biology, guidance in microdissection, helped with data analysis, and reviewed drafts of the manuscript. All authors read and approved the final manuscript.
